# Evaluating the “recovery level” of endangered species without prior information before alien invasion

**DOI:** 10.1002/ece3.863

**Published:** 2013-10-29

**Authors:** Yuya Watari, Shota Nishijima, Marina Fukasawa, Fumio Yamada, Shintaro Abe, Tadashi Miyashita

**Affiliations:** 1 Japan Forest Technology AssociationRokuban-cho 7, Chiyoda, Tokyo, 102-0085, Japan; 2 Laboratory of Biodiversity Science, School of Agricultural and Life Sciences, The University of Tokyo1-1-1 Yayoi, Bunkyo, Tokyo, 113-0032, Japan; 3 Department of Wildlife Biology, Forestry and Forest Products Research InstituteMatsunosato 1, Tsukuba, Ibaraki, 305-8687, Japan; 4 Naha Nature Conservation Office, Ministry of the EnvironmentOkinawa Tsukansha Building 4F, 5-21 Yamashita-cho, Naha, Okinawa, 900-0027, Japan

**Keywords:** Carrying capacity, invasive species management, long-term monitoring, Nansei Islands, predation, small Indian mongoose.

## Abstract

For maintaining social and financial support for eradication programs of invasive species, quantitative assessment of recovery of native species or ecosystems is important because it provides a measurable parameter of success. However, setting a concrete goal for recovery is often difficult owing to lack of information prior to the introduction of invaders. Here, we present a novel approach to evaluate the achievement level of invasive predator management based on the carrying capacity of endangered species estimated using long-term monitoring data. In Amami-Oshima Island, Japan, where the eradication project of introduced small Indian mongoose is ongoing since 2000, we surveyed the population densities of four endangered species threatened by the mongoose (Amami rabbit, the Otton frog, Amami tip-nosed frog, and Amami Ishikawa's frog) at four time points ranging from 2003 to 2011. We estimated the carrying capacities of these species using the logistic growth model combined with the effects of mongoose predation and environmental heterogeneity. All species showed clear tendencies toward increasing their density in line with decreased mongoose density, and they exhibited density-dependent population growth. The estimated carrying capacities of three endangered species had small confidence intervals enough to measure recovery levels by the mongoose management. The population density of each endangered species has recovered to the level of the carrying capacity at about 20–40% of all sites, whereas no individuals were observed at more than 25% of all sites. We propose that the present approach involving appropriate monitoring data of native organism populations will be widely applicable to various eradication projects and provide unambiguous goals for management of invasive species.

## Introduction

Invasive species are one of the major drivers for the loss of biodiversity (Sala et al. 2000), and projects for the eradication of invasive species have been undertaken worldwide to mitigate their impact. However, instances of completely successful eradication have been limited mostly to small islands or the early stages of establishments (Courchamp et al. 2003; Simberloff 2003; Clout and Russell 2006; Barun et al. 2011). Eradication activities usually require a long time and involve uncertainty regarding eradication success (Simberloff 2009; Larson et al. 2011), and continued financial support for these projects is difficult to be obtained (Park 2004; Larson et al. 2011). Evaluating the achievement level of invasive species management based on solid scientific evidence is critical for maintaining social and financial support that enables sustained eradication projects (Larson et al. 2011; Towns 2011). As a measure of achievement of eradication projects, attention should be paid not only to the decrease in the population size of the invasive species but also to the recovery of populations of native species (Lodge and Shrader-Frechette 2003). In fact, eradication or control of invasive species is one means to restore native ecosystems and biodiversity, but not a final goal in itself (Lodge and Shrader-Frechette 2003; Courchamp et al. 2011). In extreme cases, eradication of target invasive species can lead to an unexpected increase in the levels of other invasive species with undesirable outcomes in the ecosystems, for example, ecological “surprise” (Courchamp et al. 1999; Zavaleta et al. 2001; Maezono and Miyashita 2004; Rayner et al. 2007). However, setting a scientific target value can be difficult because information regarding ecosystem and community structures from prior to the establishment of invasive species is usually unavailable (Simberloff 1990). A target value can be estimated by using the status of surrogate reference sites (Mulder et al. 2009; Jones 2010; Samways and Sharratt 2010); however, these sites may not be a valid references, particularly if the target ecosystem is unique and thus not comparable with other areas (Towns 2002).

Here, we describe a new idea of estimating target values based on the carrying capacity of native organisms using long-term monitoring data. When the pressure on populations of native organisms by invasive species is released, the native species can increase in abundance. However, this rate of increase should eventually plateau by reaching carrying capacity due to limited living space or food availability. With appropriate time-series data, the carrying capacity of native species can be estimated by regressing the population growth rate on population density. Thus, the achievement of a given level of eradication or control can be evaluated explicitly when using the ratio of the current density of a native species to its carrying capacity as a target value. This method can be particularly applicable for K-strategy species such as vertebrates because these species are more likely to exhibit density-dependent population regulation (Connell and Sousa 1983; Sinclair 1989; Caughley and Sinclair 1994).

This study demonstrates the evaluation of a mongoose eradication project on Amami-Oshima Island, southwestern Japan, based on the carrying capacity of endangered native species estimated from long-term monitoring data. This island harbors a unique ecosystem including many endemic animal species. The small Indian mongoose (*Herpestes auropunctatus*: hereafter mongoose) was introduced to this island in 1979 to control a poisonous snake, Habu (*Protobothrops flavoviridis*), and the alien black rat (*Rattus rattus*) that damaged crops (Yamada and Sugimura 2004). However, the mongoose also considerably reduced the abundance of endangered species endemic to this island, such as the Amami rabbit (*Pentalagus furnessi*) and the Amami Ishikawa's frog (*Odorrana splendida*) (Sugimura et al. 2000; Watari et al. 2008). In 2000, the Ministry of the Environment, Japan, began a project to eradicate the mongoose by intensive trapping, and the estimated mongoose population size in 2011 was reduced to about 3% of the size in 2000 (Fukasawa et al. 2013). The cost of capturing a single mongoose has become very high (650,000 yen in 2011) (Ministry of the Environment Japan 2012) because the trapping efficiency in recent years has become quite low. However, until now, there has been no quantitative evidence of how native species have recovered in response to the eradication project. Here, we present the recovery levels of four native vertebrates by using monitoring data obtained at four time points spanning a 9-year period. The questions addressed are as follows: (1) Do the decreases in mongoose density result in an increased abundance of native species? (2) Do the populations of native species exhibit density-dependent growth rates to the extent that their carrying capacities can be estimated? (3) How has the mongoose eradication project succeeded when evaluated in terms of the recovery to carrying capacity of native species?

## Materials and Methods

### Study system and species

Amami-Oshima Island is the second largest of the Nansei Islands of Japan, with an area of 712 km^2^. Its climate is subtropical, with an average annual temperature of 21.5°C and annual rainfall of 2914 mm. The forested area occupies about 85% of the island, which is dominated by evergreen broadleaved trees such as *Castanopsis sieboldii* and *Schima wallichii*. High priority is given to the conservation of this island because of its high level of endemism. The World Wide Fund for Nature International ranks the forests of the Nansei Islands as one of the world's critical or endangered terrestrial ecoregions (http://wwf.panda.org/about_our_earth/ecoregions/nanseishoto_archipelago_forests.cfm). The forest on Amami-Oshima Island harbors a large number of the endemic species of the Nansei Islands.

In 1979, 30 mongoose individuals were introduced to Amami-Oshima Island to control a native poisonous pit viper, the Habu, which was a threat to local people (Tomari 1987; Sawai et al. 1999). Although the ability of the mongoose to control the Habu is equivocal, the mongoose became established in this forest, and its population in 2000 was estimated to be 5400–6800 (Fukasawa et al. 2013). Our previous work has shown that seven species of native vertebrates decreased in abundance in proximity to the release points, where mongoose density was high (Watari et al. 2008). This decrease is most likely due to predation by the mongoose, as the stomach content of trapped mongoose included these native organisms (Abe et al. 1999; Yamada et al. 2000). Thus, the Ministry of the Environment, Japan, began an eradication project in 2000, and through 2011, a total of 20,000 mongoose individuals were captured by trapping (Ministry of the Environment Japan 2012). However, complete eradication has still not been achieved, and the population size in 2011 was estimated at 40–410 (Fukasawa et al. 2013).

**Figure 1:**
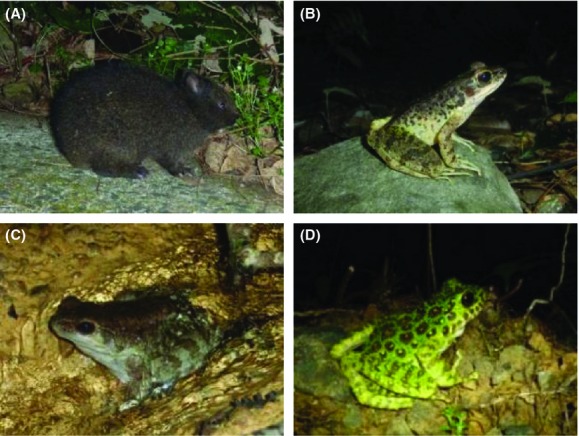
Photographs of Amami rabbit (A), Amami tip-nosed frog (B), Otton frog (C), and Amami Ishikawa's frog (D)

Here, we chose the following four endemic vertebrate species based on their conservation value and observation feasibility: the Amami rabbit (Fig. 1A), the Otton frog (*Babina subaspera*; Fig. 1B), the Amami tip-nosed frog (*Odorrauna amamiensis*; Fig. 1C), and the Amami Ishikawa's frog (Fig. 1D). All of these species are red-listed by the Ministry of the Environment Japan and inhabit only Amami-Oshima Island and adjacent islands. The Amami rabbit is the monotype species of the genus *Pentalagus*, which is considered to have diversified during the generic radiation of the leporids in the middle Miocene (Yamada et al. 2002). This rabbit has a home range of about 1 ha, presumably reaches maturity in 6–8 months (F. Yamada, unpubl. data), and is able to reproduce twice per year (Yamada and Cervantes 2005). The Otton frog is the largest Ranidae species in Japan, with a body length of 9–14 cm (Uchiyama et al. 2003), and it matures in 3 years (Iwai et al. 2006). The Amami tip-nosed frog is the smallest of the three frog species examined, with a body length of 6–10 cm (Uchiyama et al. 2003), while the Amami Ishikawa's frog has an intermediate body size of 9–12 cm (Uchiyama et al. 2003). The maturation age of the latter two species is unknown, but it is presumably similar to or less than that of the Otton frog, due to their smaller body sizes.

### Data collection and treatments

**Figure 2:**
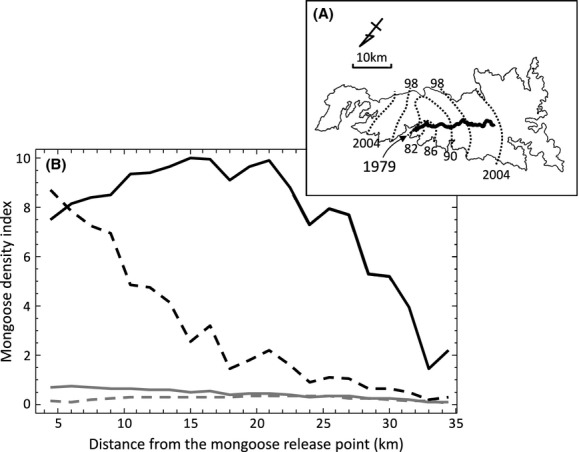
Brief descriptions of spatial and temporal dynamics of the mongoose introduced into Amami-Oshima Island. (A) Range expansion of the mongoose (dotted lines) (Environmental Agency, Kagoshima Prefecture, and Japan Wildlife Research Center 2000; Ministry of the Environment Japan 2005) and the Amami Central Forest Road (the thick line). (B) Mongoose density index along the distance from mongoose release point in the years before monitoring years (black solid line: 2002, black dashed line: 2005, gray solid line: 2008, and gray dashed line: 2010).

We conducted field surveys in the summers of 2003, 2006, 2009, and 2011. The surveys were conducted along the Amami Central Forest Road (hereafter ACF Road, 41.1 km long), which begins close to the original release point of the mongoose and leads to areas where the mongoose density was quite low (Environmental Agency, Kagoshima Prefecture, and Japan Wildlife Research Center 2000; Ministry of the Environment Japan 2005; Fig. 2A). As in the previous study, we used nighttime driving censuses for detecting animals because all four of these endemic species are nocturnal and easily found (Watari et al. 2008). The censuses were started more than 1 h after sunset. We searched for vertebrates occurring on or near the road from a car at a constant speed of about 10 km/h. We recorded species and location when we encountered them, including the call of each frog species. These surveys were conducted four times per year, in calm weather without heavy rain or strong wind. We excluded 9.6 km of paved sections of the ACF road, located at each end, from data for analyses. Data were converted to the total number of individuals observed, combined across four censuses per year per 1.5 km (hereafter referred to as “site”), as this area is larger than the home ranges of these species. The Amami rabbit has a range of 1–1.3 ha over 3 years (Yamada et al. 2000), and an adult Amami Ishikawa's frog has a range of about 1 ha per year (Nagai et al. 2011). We confirmed that these results were only slightly affected when the spatial unit was changed into 3.0 km (results not shown).

We estimated the mongoose density index at each site in each year from trapping data (Ministry of the Environment, unpubl. data). These data summarized numbers of mongoose captured and capture efforts (i.e., gross trapping days) per 1 × 1 km^2^ grid. Because CPUE (capture per unit effort) should include uncertainty and there were “blank grids” with no traps, we performed spatial and temporal smoothing of CPUE with a generalized additive model (GAM). In the GAM, we set the CPUEs as the dependent variable by using log-transformed capture efforts as an offset term and chose a negative binomial distribution with a log-link to deal with overdispersion. The independent variables were spatial locations (i.e., longitude and latitude at the center of grids) and years. We then estimated the mongoose density index at each 1.5 km site by averaging the smoothed CPUEs of meshes where the census route was traversed, weighted by the proportional length of the census route traversed by each mesh. The mongoose density thus estimated was high in early stages of the eradication program, especially near the mongoose release point, but became very low in recent years (Fig. 2B).

To determine environmental factors that might affect local carrying capacities of native species, we calculated the areas of surrounding young forests for the Amami rabbit and topographic wetness index (TWI: Wilson and Gallant 2000) for frogs. We expected that young forests at local scale might increase the carrying capacity of the Amami rabbit because herbaceous plants, a principal food for the rabbit, may be abundant there. Using published forest data (Kagoshima Prefecture 2010; Kyushu Regional Forest Office, and J. Forestry Agency 2010), we extracted forests that were 10 years or younger in 2003, 2006, and 2009 and calculated the area within six buffer sizes (15, 30, 50, 100, 250, and 500 m), which were generated along the census route. The TWI data represent the potential ability of focal ground areas to maintain surface moisture. We considered that high TWI might enhance the carrying capacities of frogs. Using altitude data from the Geospatial Information Authority of Japan, we computed TWI = log_*e*_ (*A*_*S*_/tan *β*) (where *A*_*S*_ is the specific catchment area and *β* is the slope angle) within the six buffer sizes described above. These geographical analyses were performed using ArcGIS 9.3 (ESRI 2008).

### Evaluating the recovery of endemics

We assessed the recovery of endemic species in the following three steps. The first analysis investigated whether the temporal decrease in mongoose density resulted in significantly increased densities of the four endemic species. We constructed a generalized linear mixed model (GLMM), in which the response variable was the observed number of each endemic species at each site in each year. The explanatory variables were the average of mongoose density index over all sites in the year prior to the observation year as a fixed factor and site ID as a random factor. We used a Poisson distribution for the model's error term in the Amami Ishikawa's frog, while a negative binomial distribution in the Amami rabbit, the Amami tip-nosed frog, and the Otton frog to deal with overdispersion. We calculated *p*-values on the basis of likelihood ratio tests. In this analysis, we excluded sites where no individuals of each endemic species were observed during 9 years. As these sites were located near the original mongoose release point, we assumed that endemic species had been locally extirpated by long-term high predation pressure from the mongoose and still had not recolonized.

Second, we estimated the carrying capacities of endemics at each site. Although carrying capacities are generally estimated by identifying a density at which the population growth rate is zero (Sibly et al. 2007), such a method might underestimate carrying capacities of native species in our study due to the presence of mongoose, even at low densities. Thus, we used the following equation including mongoose predation as well as logistic growth to estimate the carrying capacity in the absence of mongoose:


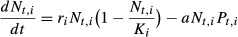
(1)

where *N*_*t,i*_ and *P*_*t,i*_ are the densities of native species and mongoose, respectively, at site *i* in time *t*, *r*_*i*_ and *K*_*i*_ are the intrinsic growth rate and the carrying capacity, respectively, of native species at site *i*, and *a* represents the attack rate of mongoose on native prey. Here, we used a linear functional response by the following two reasons. First, the mongoose is a generalist predator consuming numerous prey items and therefore unlikely to exhibit a saturating functional response to a particular prey species. Second, this simple function allows us to analytically integrate equation (1) under the assumption of a fixed mongoose density, which facilitates parameter estimations. The population growth rate of native prey species in time interval Δ*t*, obtained by analytical integration, is described as follows:


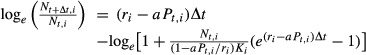
(2)

This equation corresponds to a transformed logistic growth in which both intrinsic growth rate and carrying capacity are lowered by mongoose predation: 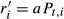 and 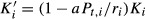. The first and second terms of the right side in equation (2) represent, respectively, density-independent and density-dependent population growth of native species. The time intervals analyzed were from 2003 to 2006, 2006 to 2009, and 2009 to 2011 (i.e., Δ*t* = 2 or 3 years). We used the average mongoose density index between focal successive years at each site. For instance, when the time interval was from 2003 to 2006, the algebraic mean of mongoose density indices in 2003, 2004, and 2005 was used. We considered that the local carrying capacity could be affected by environmental factors (young-forest area for the Amami rabbit; TWI for frogs): *K*_*i*_ = *K* + *β* env_*i*_, where *K* is the average carrying capacity; *β* is the coefficient of an environmental factor; and env_*i*_ is the explanatory variable of an environmental factor, whose values were scaled by mean and SD. For the Amami rabbit, we used the area of young forests in the first years of time intervals analyzed (i.e., 2003, 2006, and 2009). To avoid pseudoreplication, we included a random site effect for *r*: *r*_*i*_ = *r* + [site]_*i*_, where *r* is the average intrinsic growth rate and [site]_*i*_ is a random coefficient.

To explore the occurrence of density dependence and to estimate local carrying capacity, we compared the AIC of 8 candidate models with and without density dependence, or with and without local environmental factors for carrying capacity (Table 1). The first model has no density dependence (with no second term in the right side in equation (2)), the second model includes density dependence with no environmental factors, and the remaining six models have density dependence with environmental factors and different buffer sizes. All of the candidate models included the effect of mongoose predation, because our aim was to estimate carrying capacity of native organisms in the absence of mongoose. To avoid log (0), we added 0.5 to both the denominator and the numerator of the left side in equation (2). We investigated the sensitivity to added value and found that carrying capacities estimated by adding 0.1 and 1.0 were 92–98% and 102–104%, respectively, of those estimated by adding 0.5. For each candidate model, we estimated parameters using a nonlinear mixed-effects model with a Gaussian distribution. Estimated carrying capacity from all surveys was divided by four to obtain an average of individuals observed per survey. In this analysis, we excluded the following time-transition data: 0 to 0, 1 to 0, and 2 to 0, because “0 to 0” has no information, and “1 to 0” and “2 to 0” could strongly reflect demographic stochasticity under low densities; inclusion of these data could lead to underestimation of deterministic population growth rate and carrying capacity. Notice that including these data did not change the qualitative conclusion regarding the significance of density dependence (results not shown).

**Table 1:** AIC of candidate models for predicting the population growth rate of four endemics.

Fixed effects^1^	Amami rabbit	Amami tip-nosed frog	Otton frog	Amami Ishikawa's frog
*r*, *a*	121.56	112.04	67.96	69.69
*r*, *K*, *a*	70.00	92.52^2^	38.68^2^	45.16
*r*, *K*, *a*, *β*_*15*_	62.19	93.79	40.18	41.82
*r*, *K*, *a*, *β*_*30*_	61.10^2^	93.43	39.57	40.09
*r*, *K*, *a*, *β*_*50*_	61.87	94.30	39.47	40.88
*r*, *K*, *a*, *β*_*100*_	65.12	94.50	39.37	39.60^2^
*r*, *K*, *a*, *β*_*250*_	65.48	94.50	42.44	44.48
*r*, *K*, *a*, *β*_*500*_	64.42	94.40	38.70	45.42

AIC is an information criterion for evaluating goodness of prediction based on the deviance (*D*) and the number of parameters (*k*): AIC = *D* + 2*k*. Lower AIC means better model for prediction.

1 *r*, *a*, *K*, and *β* are the intrinsic growth rate, the mongoose attack rate, the carrying capacity, the coefficient of environmental factor, respectively. Subscripts of *β* represent buffer sizes (*m*).

2 The best model (with the lowest AIC).

Lastly, we evaluated the recovery of native species using estimated carrying capacity. We regarded endemic species to have recovered at the site level if the observed number reached the estimated carrying capacity. All statistical analysis was performed using statistical software R version 2.13.0 (R Development Core Team 2011). We used the R packages of “mgcv” (Wood 2011) for GAM, “glmmADMB” (Skaug et al. 2011) for GLMM, and “nlme” (Pinheiro et al. 2011) for nonlinear mixed-effects model.

## Results

**Figure 3:**
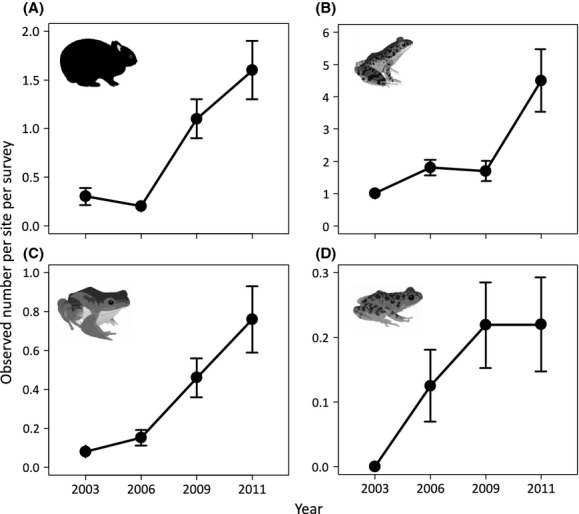
Temporal changes in numbers of four species observed per site per survey (mean ± SE). (A) Amami rabbit, (B) Amami tip-nosed frog, (C) Otton frog, and (D) Amami Ishikawa's frog.

The numbers of all four endemic species have clearly increased as the mongoose eradication project began (Fig. 3). The likelihood ratio tests showed that the average mongoose density index significantly affected the density of these four species (*P* < 10^−3^ for the Amami rabbit, the Amami tip-nosed frog, and the Otton frog; *P* < 10^−5^ for the Amami Ishikawa's frog). The site effect was significant for the Amami tip-nosed frog (*P* < 10^−4^) and the Amami Ishikawa's frog (*P* < 10^−2^), but not for the Amami rabbit (*P* = 1) or the Otton frog (*P* = 0.11).

**Figure 4:**
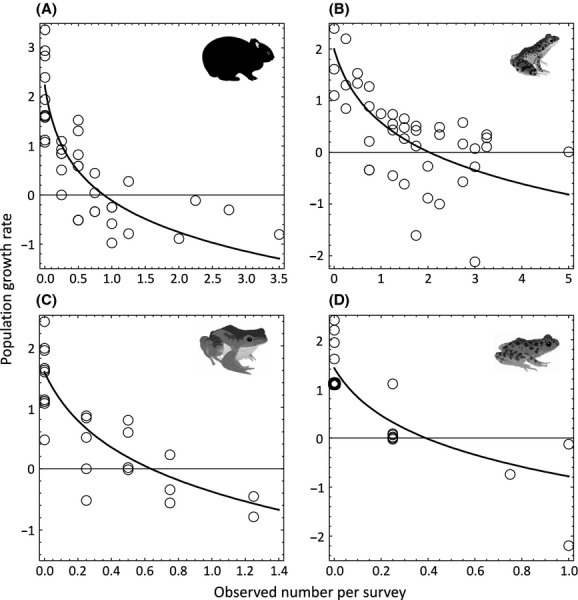
Density-dependent growth in four species. (A) Amami rabbit, (B) Amami tip-nosed frog, (C) Otton frog, and (D) Amami Ishikawa's frog. Circles represent population growth rates observed in monitoring intervals. Black curves indicate the population growth rates during average monitoring span where the mongoose density is zero, predicted from the best models.

**Figure 5:**
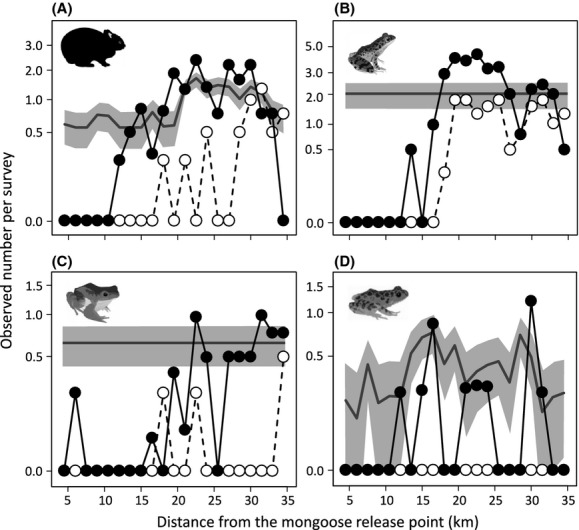
Comparison of observed numbers with the carrying capacities estimated from the best models. (A) Amami rabbit, (B) Amami tip-nosed frog, (C) Otton frog, and (D) Amami Ishikawa's frog. Observed numbers per survey in 2003 (white circles and dashed lines) and 2011 (black circles and solid lines) are plotted along the distance from mongoose release point. Gray thick lines and shadowed areas represent the mean and 95% confidence interval, respectively, of carrying capacities. Spatial variations in estimated carrying capacities of Amami rabbit and Amami Ishikawa's frog are due to the effects of young-forest area and TWI, respectively. The variation for the Amami rabbit is based on the area of young forests in 2009.

For all endemic species, models including carrying capacity (*K*) had much lower AIC than the competing model without carrying capacity (Table 1). This indicates that density-dependent population growth is actually occurring in the field (Fig. 4). The 95% confidence interval for *K* was relatively small for the Amami rabbit (48% of the estimated *K* in the best model), the Amami tip-nosed frog (58%), and the Otton frog (65%), but large for the Amami-Ishikawa's frog (115%) (Fig. 5).

In addition to the carrying capacity, the best model included an environmental variable in Amami rabbit and Amami Ishikawa's frog, and models without the variable had much lower performance in terms of AIC values (Table 1). In the Amami rabbit, surrounding young forests had a positive coefficient (*β* = 0.33 in the best model), meaning that young forest appeared to enhance local carrying capacity of this species. In the Amami Ishikawa's frog, TWI had a positive coefficient (*β* = 0.19), implying higher local carrying capacity in sites with more ground surface moisture. As shown in Fig. 5, these effects were reflected in the spatial variation of the local carrying capacity in the two species. For the Amami tip-nosed frog and the Otton frog, however, TWI little explained the variation in local carrying capacity because AIC values were similar between models with and without TWI (Table 1).

The number of sites that had recovered increased significantly from 2003 to 2011 for all species examined (Fig. 5). In 2003 when mongoose density was still high, 10% (two of 21) sites that were far from the original mongoose release point remained close to the carrying capacity for the Amami rabbit, while there were no such sites for the three frog species. In 2011 when mongoose density was quite low, populations of the Amami rabbit and the Amami tip-nosed frog had recovered at 38% (eight of 21) sites, while populations of the Otton frog and the Amami Ishikawa's frog had recovered at 19% (four of 21) sites. However, no individuals were still observed at 29–62% (6–13 of 21) sites, most of which were located near the release point except for the Amami Ishikawa's frog.

## Discussion

The small Indian mongoose is one of the most invasive species in the world, and thus, eradication programs are being implemented on many Pacific islands other than Amami-Oshima Island (Barun et al. 2011). Earlier studies evaluated the impacts on native organisms by examining stomach content of mongoose or by spatially correlating the distributions of mongoose and native species (Watari et al. 2008; Barun et al. 2010). However, these types of information are insufficient to demonstrate causality because consumption of a particular prey by a predator does not necessarily imply the existence of population-level effects, and a negative spatial association between predator and prey might simply reflect environmental gradients differentially affecting each species (Park 2004). To prove causal relationships, we need to examine the positive responses of populations of native species after the removal of invasive predators (Park 2004; Fukasawa et al. in press).

Here, we provided clear evidence that the decrease in mongoose density due to the eradication project resulted in increased populations of four native species. A fundamental assumption in our study is that the observed number of native individuals is proportional to the actual number of individuals. However, change in detection probability due to behavioral change in response to mongoose density might also affect the number of observed individuals of native species. We consider this effect is unlikely to be important because the number of individuals observed close to the original mongoose release point did not increase even though the mongoose density became quite low in the past several years. This could be due to dispersal limitation from source habitats, as will be explained later in detail. This evidence thus strongly suggests the presence of population-level impacts of the mongoose on native species rather than decreased detection probability of individuals.

Another important finding is that a density-dependent decrease in population growth rate was detected for all of the native species examined, which allowed us to estimate their carrying capacities. The strength of our approach is that we used a population growth model that combined effects for logistic growth and mongoose predation. As native species under predation pressure from invasive species could exhibit an equilibrium density that is lower than the true carrying capacity without invasive species, incorporating the term for mongoose predation into the model is essential. Without considering the predation effect, carrying capacity would have been underestimated.

The carrying capacities of three native species (the Amami rabbit, the Amami tip-nosed frog, and the Otton frog) other than the Amami Ishikawa's frog seem to be well estimated, because the lower limits of the confidence intervals were far above zero, lying at around 70% of the mean values. Thus, the carrying capacities presented here can be used as target values for restoration of native species, except for the Amami Ishikawa's frog. Nevertheless, non-negligible uncertainties arising for multiple reasons, including observation and process errors, are involved. Some degree of observation error at their low densities is inherently unavoidable because the target organisms threatened by the mongoose are nocturnal and endangered. Also, asymmetric immigration rates between low- and high-density areas might cause a process error that underestimates local carrying capacities. For instance, some of the population declines found in high-density sites may reflect, in part, fewer immigrants from surrounding low-density sites, resulting in a bias toward lower equilibrium density compared with the actual one. Including dispersal between sites makes models more complex, making it difficult to estimate population parameters with limited time-series data, thus we did not employ this approach here.

For all native species examined, local sites that remained close to the carrying capacity were rarely found in 2003, but increased to 19–38% of sites by 2011, some of which exhibited even higher densities than the carrying capacity. This indicates strong support that the eradication project was very successful in some areas. However, the population density of native species in some sites still remained at very low levels, especially those located near the original release site of the mongoose, despite very low mongoose density for the last several years. At these sites, long-term strong predation pressure by the mongoose may have locally extirpated native species, and a limited amount of dispersal from source habitats, in which mongoose density had been low prior to beginning mongoose eradication (i.e., areas farther from the mongoose release site), is likely to retard native species recovery. Dispersal limitations appear to be most severe for the Otton frog, followed by the Amami tip-nosed frog, and lowest for the Amami rabbit. These differences may be due to dispersal ability, reproductive potential, or both. The Otton frog is the largest Ranidae species in Japan, taking three or more years to mature (Iwai et al. 2006), and it thus appears to have a low population growth rate. On the contrary, the Amami rabbit reaches maturity presumably in 6–8 months (F. Yamada, unpubl. data) and can reproduce twice per year (Yamada and Cervantes 2005), suggesting a relatively high population growth rate. These differences in life-history characteristics may have contributed to the differences in observed population density patterns. In any case, recolonization will be key to successful restoration of native species on Amami-Oshima Island, and thus, continuous mongoose trapping and endemic monitoring efforts are essential.

Most eradication programs for invasive species are aimed at complete eradication of target species, but financial support for them has often been terminated or greatly reduced when capture efficiency became low (Howald et al. 2007). Such projects were then regarded as a failure, and judged not to be worth continued support. However, if native communities or ecosystems are to be restored by reducing populations of invasive species, failure to completely eradicate invasive species does not mean the project has failed. Scientific evidence that native species have recovered to carrying capacity in some sites but not in others provides a solid rationale for low-density management of invasive species as an alternative strategy to complete eradication and thus continued support of control programs. There has been a conceptual issue of using the carrying capacity of species with conservation concern as a target value for assessing success or failure of management actions (Sanderson 2006). To our knowledge, however, our study is the first to empirically demonstrate the recovery of native species to carrying capacity upon invasive species management. The absence of such evidence to date is probably due to an absence of long-term monitoring data that enable novel methods of statistical estimation of the carrying capacity of conservation targets. We suggest that, if proper time-series data are available, our method is widely applicable to native organisms especially with K strategies, such as mammals, birds, and fish (Sibly et al. 2007), because these organisms are by definition more likely to exhibit density dependence in nature. It is thus crucial to incorporate native organism monitoring systems into invasive species management programs and to provide the public with information regarding the progress of these programs with respect to the target value (carrying capacity) of native species. This will encourage continued financial support for eradication programs in the long term.
